# Study protocol for a pre/post study on knowledge, attitudes and behaviors regarding STIs and in particular HPV among Italian adolescents, teachers, and parents in secondary schools

**DOI:** 10.3389/fpubh.2024.1414631

**Published:** 2024-08-19

**Authors:** Laura Brunelli, Francesca Valent, Manola Comar, Barbara Suligoi, Maria Cristina Salfa, Daniele Gianfrilli, Franz Sesti, Vincenzo Restivo, Alessandra Casuccio, ESPRIT Study Collaboration Group, Silvia Gazzetta, Lorenza Driul, Andrea Isidori, Patrizia Ferro, Nicoló Piazza, Palmira Immordino, Giuseppina Capra, Teresa Fasciana

**Affiliations:** ^1^Dipartimento di Medicina, Università degli Studi di Udine, Udine, Italy; ^2^SOC Accreditamento, Qualità e Rischio Clinico, Azienda Sanitaria Universitaria Friuli Centrale, Udine, Italy; ^3^SOC Igiene e Sanità Pubblica, Dipartimento di Prevenzione, Azienda Sanitaria Universitaria Friuli Centrale, Udine, Italy; ^4^Diagnostica Avanzata Microbiologica Traslazionale, IRCSS Burlo Garofolo, Trieste, Italy; ^5^Dipartimento Universitario Clinico di Scienze Mediche Chirurgiche e della Salute, Università degli Studi di Trieste, Trieste, Italy; ^6^Dipartimento Malattie Infettive, Centro Operativo AIDS, Istituto Superiore di Sanità, Rome, Italy; ^7^Dipartimento di Medicina Sperimentale, Università Sapienza di Roma, Rome, Italy; ^8^Dipartimento di Medicina, Università Kore di Enna, Enna, Italy; ^9^Sezione di Igiene, Dipartimento PROMISE, Università di Palermo, Palermo, Italy; ^10^SOC Ostetricia e Ginecologia, Azienda Sanitaria Universitaria Friuli Centrale, Udine, Italy; ^11^Sezione di Microbiologia, Dipartimento PROMISE, Università di Palermo, Palermo, Italy

**Keywords:** prevention, sexually transmitted infections, HPV, youth, adolescents, parents, school

## Abstract

Sexually transmitted infections (STIs) are one of the most important issues related to sexual and reproductive health, as it is estimated that more than 1 million new infections are acquired every day worldwide and data on the prevalence and incidence of these infections, especially among young people, are increasing. Nevertheless, there are some knowledge and behavioral gaps, and young people need more support from their school and family network to protect themselves and their peers. Therefore, we have designed a multicenter prospective intervention study involving public lower and upper secondary school students, their parents and teachers (ESPRIT). The intervention will take place in the school year 2023–2024, where students will meet with experts and be involved in peer education, while adults (parents and teachers) will participate in distance and face-to-face trainings. All target groups will complete KAP (knowledge, attitudes, practice) questionnaires before and after participating in the intervention to measure its effectiveness. The results of this study will help to assess and improve the level of knowledge of lower and upper secondary school students, parents and teachers about STIs and HPV in particular, raise awareness of sexual and reproductive health issues, including vaccination, among lower and upper secondary school students and their families, and evaluate the effectiveness of these interventions in terms of improving knowledge and changing attitudes and behaviors. The study protocol has been approved by the Regional Unique Ethics Committee of Friuli Venezia Giulia (CEUR-2023-Sper-34). The project is being carried out with the technical and financial support of the Italian Ministry of Health—CCM.

## Introduction

Sexual health, as defined by the World Health Organization (WHO), is a fundamental goal that must be pursued to ensure that everyone can have safe sexual experiences that are free from coercion, discrimination, and violence. This right, which applies to both women and men, must also apply to adolescents and therefore requires full and free access to health information and services, as well as the provision of tools for the prevention of sexually transmitted infections (STIs) and safe contraceptive methods (1). In particular, the definition of comprehensive sexuality education (CSE) proposed by UNESCO in 2018 as a process based on a teaching and learning curriculum that integrates the cognitive, emotional, physical, and social aspects of sexuality is relevant to this topic. The aim of CSE is to equip children and young people with knowledge, skills, attitudes and values that enable them to recognize their own health, well-being and dignity, to develop respectful social and sexual relationships, to consider how their choices affect their own well-being and that of others, and to understand and protect their rights throughout their lives (2).

The contribution of sexual and reproductive health (SRH) to global and individual health was recently highlighted by the inclusion of this topic in the Sustainable Development Goals, in particular target 3.7 which aims to ensure universal access to sexual and reproductive health services, including family planning, information and education, and the integration of reproductive health services into national policies and programs by 2030 (3). Since 2000, some SRH indicators have improved at the European level, such as the decline in perinatal mortality and abortion rates (4), but many challenges remain. STIs are indeed one of the most important issues in SRH, as it is estimated that more than 1 million new infections are acquired every day worldwide. These new infections are caused by pathogens such as *Trichomonas vaginalis, Chlamydia trachomatis, Neisseria gonorrhoeae, and Treponema pallidum*, but also by viral pathogens such as herpes simplex virus, papillomavirus, and HIV, which also have worrying numbers (5). In addition, there are other pathogens that are not strictly considered sexually transmitted but are also transmitted through sexual contact, such as Shigella, Salmonella, *Giardia lamblia*, HAV (6), Zika (7), and other arboviruses (8) and monkeypox (9). A closer analysis of European data on STIs shows a less reassuring trend: HIV incidence, for example, has doubled in the last two decades (4, 10), and *Chlamydia trachomatis* infection remains the most common bacterial cause of STIs, especially among young people aged 15–24 (11).

At the Italian level, the number of people with a confirmed STI increased by around 23 percent between 2000 and 2019, according to data from two sentinel STI surveillance systems. Chlamydia cases increased by 33 percent in 2019 compared to 2017, and gonorrhea cases even doubled from 2014 to 2019 (12). This disease burden is exacerbated by the fact that some pathogens that cause STIs also have oncogenic potential for both sexes: HPV is responsible for a large proportion of cervical and anogenital cancers, and can cause oropharyngeal cancer, while HBV is the leading cause of liver cancer worldwide (13, 14). In Italy the prevalence of chlamydia in girls aged 15–24 years (7.5%) is three to four times higher than in subsequent age groups: a worrying fact that deserves mention (15). Another emerging problem in this area is the increase in infections caused by multidrug-resistant pathogens, particularly in relation to third-generation cephalosporins, which is becoming a major public health concern due to the few therapeutic alternatives and the lack of a vaccine (16, 17). In addition, scientific evidence of ‘HPV-related’ co-infection with *Chlamydia trachomatis*, an asymptomatic STI that can have serious consequences for reproductive life, including infertility, must also be considered in adolescents (18–20), as well as worrying data on HPV-HIV co-infection (21, 22). In the European Region, cervical cancer remains the second most common cancer in women aged 15–44 years and, despite advances in screening and vaccination, nearly 27,000 women die from the disease each year (23). In Italy, cervical cancer is the fifth most common cancer in young women and 2,300 new cases were registered in 2017, killing 494 women despite advances in research (24). Despite the alarming burden of STIs in adolescents, which is considered one of the top 20 causes of disability-adjusted life years (DALYs) in children under 19 years of age (25), and the high susceptibility of young people to long-term oncologic and reproductive complications due to biological, behavioral, and cultural characteristics typical of this age (26), knowledge and risk perception among adolescents unfortunately appear to be limited (27–29). Although, Italy was the first European country to introduce universal HPV vaccination for adolescent females and males in 2017, offered free and actively at the age of 12 (30), recent national vaccination data are not reassuring (31) and, moreover, the vaccination coverage for 2020 has dropped significantly compared to 2019 (32), probably due to the impact of the pandemic on health services (33).

In response to the recent WHO programmatic document (34), the latest National Prevention Plan 2020–2025 specifically addresses SRH issues under two of the six macro-objectives (35). According to the plan, the school is the place where health promotion should take place as part of a continuous and integrated pedagogical approach throughout the school career; therefore, better interaction between schools and local health services is needed to enable effective integration of specific competences. Compared to other European countries, Italian adolescents are still much less exposed to SRH education (27, 36) than in other countries (37). Some activities in the national context are proving to be effective, such as the already launched EduForIST project, which focuses on the development of technical and practical tools for the implementation of CSE and training in the school context (38). However, initiatives continue to be implemented inconsistently across the country, often as individual projects without a fixed and structured place in planning as true systematic processes (39). Although there is a clear need for STI prevention programs for young people, the sensitive content, the difficulty of determining the target age, the complexity of creating appropriate and standardized teaching materials and a certain reluctance on the part of parents and teachers have so far hindered the inclusion of these educational modules in the curricula.

For these reasons, in 2022 the Italian Ministry of Health funded a new CCM aimed at the promotion, development, implementation and evaluation of educational activities in the areas of sexuality, emotional relationships and prevention of sexually transmitted infections in the national school environment. To respond to this call, the partners developed a study project entitled “Education in lower and upper secondary school and support of the network of adolescents reference persons for the prevention of HPV and other sexually transmitted infections (ESPRIT).”

## Objectives

The ESPRIT study aims to: (1) assess the level of knowledge, attitudes and behaviors regarding STIs and in particular HPV among secondary schools adolescents, teachers, and parents; (2) develop an information and education program for adolescents, parents and teachers regarding STIs and in particular HPV; (3) evaluate the effectiveness of this program among the same three target groups (i.e., adolescents, parents and teachers) regarding knowledge and attitudes toward STIs and in particular HPV.

## Methods and analysis

Participating centers (all Italian): Friuli Centrale Healthcare University Trust, Udine, Italy; Advanced translational microbiology diagnostics, IRCSS Burlo Garofolo, Trieste, Italy; National AIDS Unit, Department of Infectious Diseases, Istituto Superiore di Sanità, Rome; Department of Experimental Medicine, Sapienza University of Rome, Rome; PROMISE Department, University of Palermo, Palermo.

### Selection of schools

Inclusion criteria: public lower and upper secondary schools that have agreed to participate in the project and represent both urban and extra-urban areas in the provinces of Rome/Frosinone, Palermo, and Udine (Italy). Exclusion criteria: public secondary school classes already participating in other information and education projects on STIs/HPV prevention or on SRH in general.

The schools are selected by the participating centers in collaboration with the local health authorities based on the inclusion and exclusion criteria mentioned above. The selected schools will first be contacted by the regional contact person by letter and/or e-mail to introduce the project in general and to inquire about the willingness of the institutions to participate. To this end, the head teachers are provided with background information and the objectives to be pursued as well as the modalities and timetable of the intervention.

### Involvement of participants in the selected schools

Inclusion criteria: adolescents attending a lower secondary school (grade II, year 7) or upper secondary school (grade II or III, year 10 or 11); parents of adolescents attending the same school; teachers of adolescents attending the same school. Exclusion criteria: insufficient understanding of the Italian written language; non-consent to participate; inability to give informed consent.

### Intervention

The intervention proposed in the ESPRIT study, to be carried out in the 2023–2024 school year, is aimed at adolescents in lower secondary education (grade II) and upper secondary education (grade II or III), as well as their parents and teachers.

Given the three different target groups, the proposed intervention consists of the following elements:

(1) Training course for adolescents through:
(1A) Conducting sessions in lower secondary schools by a multidisciplinary team of experts on knowledge, attitudes, behaviors, and information on STIs and HPV (three sessions of 1–2 h during school hours). This intervention was considered more age-appropriate for students, as reported by colleagues from previous experience (40).
(1B) Conducting peer education in upper secondary schools on knowledge, attitudes, behaviors, and information on STIs and HPV. As reported in the literature, there is good evidence of conducting peer education with adolescents and young people on this topic and its effectiveness in improving knowledge and attitudes (41, 42) and improving access to health services (43). To conduct peer education, at least two young volunteers will be identified in each school, supervised by a multidisciplinary team of experts to prepare the contents and modalities according to which the peer meetings will be conducted. The additional hourly commitment of students to the project will be encouraged, when possible, through the recognition of educational credits. The peer education meetings with peers will take place during school hours at times agreed with the school principal and the teachers of the classes involved.
(2) Information and training courses for teachers will be provided remotely through MOOCs available on the project website. Additional face-to-face/remote meetings will be held to provide further information and tools for adult-adolescent relationships on the specific topic of STIs and HPV. Participation in the activity will be duly recognized through a certificate of participation and, where possible, continuing education credits.
(3) An information and education course for parents will be offered remotely through a MOOC that will be made available on the project website. Additional face-to-face/remote meetings will be held to provide further information and tools for adult-adolescent relationships on the specific topic of STIs and HPV.

The multidisciplinary team of experts implementing the intervention (i.e., session structure, materials, content, activities) may consist of the following professionals depending on local availability: psychologist or sexologist, gynecologist, andrologist, dermatologist, public health expert, microbiologist, infectious disease specialist, health assistant, midwife, pediatrician. The expert teams of all participating centers will continuously monitor the activities and exchange experiences during the project implementation by organizing local and national meetings.

### Mode and time of data collection

Data will be collected on the number and type of schools participating in the project, the number of information/training sessions and associated participants, the number of students involved as peer educators and the number of peer meetings held.

To measure the effectiveness of these information and education measures, questionnaires will be completed before and after participation in the intervention to assess knowledge, attitudes, and behavior regarding STIs and HPV. The same basic questionnaire was adapted for each participant category (lower secondary school students, upper secondary school students, teachers, parents) to make the questions understandable for the target groups and thus facilitate participation and tested with representative groups. The questionnaires for students, teachers and parents are available as Supplementary material. Participation in the project is completely free of charge for schools and individuals. The questionnaires are only collected with the informed consent of the adult participants themselves, or the parents in the case of minors, as well as the consent of the minors themselves.

The questionnaires have been developed on the basis of discussions and comparisons between professionals from different fields (public health, microbiology, epidemiology, gynecology, andrology) with the aim of using appropriate language for adolescents, on the one hand and adults on the other; identifying the most relevant aspects of the different topics in order to reduce the length of the questionnaires; taking into account and respecting the potentially different views and sensitivities of the users; making the interface with the user simple and intuitive, to name but a few. The questionnaires are made available online on a platform provided by the National Institute of Health (ISS, Istituto Superiore di Sanità). After obtaining informed consent, each participant will be assigned a randomly generated unique 6-digit alphanumeric code, which is entered at the beginning of the questionnaires before and after the intervention. The questionnaires will then be pseudo-anonymized and the key to the participant’s data will remain accessible only to the researchers, who retain the consent forms and can use this information in the event of posthumous withdrawal of consent by the participant. The data collection platform has been designed by a team of information technology experts to be encrypted, secure, privacy-focused, compliant with applicable legal standards, robust, protected, fortified and secure. The database will be centralized. Data collection will start in 2023 and end before December 2024.

### Sample size

The response to question no.3 regarding knowledge that HPV is a sexually transmitted infection will be used as the outcome of interest. Assuming that 5% of adolescents who answered both the pre-intervention questionnaire and the post-intervention questionnaire switch between correct and incorrect answer, and 25% switch between incorrect and correct answers, and with an intraclass correlation coefficient (ICC) of 0.1, with an average cluster of 20 subjects, applying a continuity correction, the study requires at least 169 adolescents answering both the pre- and post-intervention questionnaires to obtain 80% statistical power of and accept and alpha error of 5% to find difference of 0.20 between the mismatched proportions (at least 9 clusters of 20 subjects) (44).

### Data analysis plan

Data collected in the pre-intervention questionnaire will be analyzed using frequency distributions for categorical variables and measures of central tendency (e.g., mean, median) and of dispersion (e.g., standard deviation) for continuous numerical variables. The data collected in the pre- and post-intervention questionnaires will be compared using the McNemar test, considering only those subjects who participated in both surveys, with particular interest for those subjects who gave an incorrect answer before the intervention and a correct answer after the intervention and vice versa. The adolescent group is considered the main target group. In order to reduce the bias that could result from the fact that the sample selection is not random, the characteristics of the school (urban/extra-urban, secondary/secondary school, north/center/south Italy) and the characteristics of the students (country of birth, gender, parents’ education level) will be weighted using propensity scores. In order to determine differences between socio-demographic characteristics in the knowledge values of the questionnaire, we will use parametric tests such as the *t*-test or ANOVA, provided their assumptions—normality (assessed with the Shapiro–Wilk test) and homoscedasticity (assessed with the Levene test)—are met. If these assumptions are not met, non-parametric tests such as the Mann–Whitney U test or the Kruskal-Wallis test will be used. The effect sizes (Cohen’s d for *t*-tests, Eta-square for ANOVA) will be given to determine the extent of the differences. The *p*-values (alpha) and, if possible, the power of the test (1-beta) will be given for the tests. For non-parametric tests, Monte Carlo simulations or bootstrapping techniques will be used to approximate these values. In addition, a regression model (or generalized linear model, GLM) will be fitted to estimate the coefficients of various student factors (e.g., region, area, gender, country of birth, parents’ level of study). This will provide further insight into the relationships and potential effects of these factors within the study. The analyses will be conducted using R software, version 4.4.0 (45).

## Discussion

As far as we are aware, this is the first multicenter study at national level aimed at testing the effectiveness of a school-based project for the prevention of STIs and HPV in adolescents in lower and upper secondary education. It is also one of the first studies to examine the practical implementation of peer education on this topic at a national level. We therefore plan to compare our results in terms of efficiency and effectiveness with the results of studies conducted in similar settings in the past, such as those by Icardi et al. (40) and Zizza et al. (46). Moreover, the results of this project will be important to inform and guide future public health interventions, HPV vaccination campaigns, health promotion and disease prevention strategies. The decision to consider both urban and extra-urban areas will provide more information on context-specific opportunities and strengths, together with the availability of experience from implementation in different Italian regions, that might be associated with specific characteristics that need to be considered, as there is no one size fits all. The continuous comparisons and exchange of experiences between the multiprofessional expert teams of the different regions will help to develop a content program and a set of tools relevant for any other public health institution interested in implementing the same activities in their local context.

## Ethics and dissemination

The reading of the questionnaire responses, their collection in a special database and the subsequent data analysis are limited to the working group. The data will be presented in summarized form to make it impossible to trace the identity of the participants. The collected data will therefore be used for the preparation of reports and publications while maintaining anonymity. The data will be kept for as long as it is useful for the purposes of the study. Each participant will receive information about the purpose of the study and the methods of data collection and intervention. The data collection platform ensures full compliance with the data protection regulations currently in force at EU level (EU-GDPR). The study protocol has been approved by the Regional Unique Ethic Committee of Friuli Venezia Giulia (CEUR-2023-Sper-34). The project is being carried out with the technical and financial support of the Italian Ministry of Health - CCM. The results of the study will be made available to the scientific community, relevant stakeholders, and the public through publications, public communications, and social media posts.

## Ethics statement

ESPRIT study design: multicenter, prospective, interventional, study. The studies involving humans were approved by the Regional Unique Ethics Committee of Friuli Venezia Giulia (CEUR-2023-Sper-34). The studies were conducted in accordance with the local legislation and institutional requirements. Written informed consent for participation in this study was provided by the participants’ legal guardians/next of kin.

## Author contributions

LB: Conceptualization, Investigation, Methodology, Project administration, Supervision, Writing – original draft, Writing – review & editing. FV: Conceptualization, Validation, Writing – review & editing, Data curation, Investigation. MC: Investigation, Writing – review & editing, Supervision. BS: Writing – review & editing, Data curation, Investigation, Methodology, Supervision. MCS: Investigation, Writing – review & editing, Data curation, Methodology. DG: Data curation, Methodology, Writing – review & editing, Supervision, Validation. FS: Investigation, Methodology, Writing – review & editing. VR: Methodology, Writing – review & editing, Investigation. AC: Supervision, Writing – review & editing, Investigation, Methodology.

## ESPRIT Study Collaboration Group

Silvia Gazzetta, Lorenza Driul, Andrea Isidori, Patrizia Ferro, Nicoló Piazza, Palmira Immordino, Giuseppina Capra, and Teresa Fasciana.
