# Erratum: Study protocol for a pre/post study on knowledge, attitudes and behaviors regarding STIs and in particular HPV among Italian adolescents, teachers, and parents in secondary schools

**DOI:** 10.3389/fpubh.2024.1518632

**Published:** 2024-11-05

**Authors:** Frontiers Production Office

**Affiliations:** Frontiers Media SA, Lausanne, Switzerland

**Keywords:** prevention, sexually transmitted infections, HPV, youth, adolescents, parents, school

Due to a production error, the names of the ESPRIT Study Collaboration Group (Silvia Gazzetta, Lorenza Driul, Andrea Isidori, Patrizia Ferro, Nicoló Piazza, Palmira Immordino, Giuseppina Capra, and Teresa Fasciana) were erroneously omitted in the article.

The publisher apologizes for this mistake. The original version of this article has been updated.

