# Supplementary material for: Study protocol for a pre/post study on knowledge, attitudes and behaviors regarding STIs and in particular HPV among Italian adolescents, teachers, and parents in secondary schools
Source: Front Public Health. 2024 Aug 19;12:1414631. doi: 10.3389/fpubh.2024.1414631 (PMC11366568; doi:10.3389/fpubh.2024.1414631)
Supplement: Supplementary file 1 [file Table_1.DOCX]

**ESPRIT study - Questionnaire for adolescents**

Participant ID code: ______________

*Knowledge section*

1. Have you ever heard of sexually transmitted infections? Yes / No

2. How can sexually transmitted infections be transmitted? Through: (multiple answers possible)

Options: Handshake / Deep kissing / Contact between genitals / Vaginal intercourse / Anal intercourse / Oral intercourse / Use of sanitary facilities in communal/public bathrooms / Use of contaminated objects (e.g., towels) / Blood transfusions / Exchange of syringes / Other (specify..........................) / Don't know

3. In your opinion, can the following infections/diseases be transmitted through sexual intercourse? Yes / No / Don't know

Diseases: Viral hepatitis type B / Syphilis / Rubella / Chlamydia / Influenza / AIDS / Mediterranean anemia / Gonorrhea / Viral hepatitis type A / Papillomavirus infections / Diabetes / Genital herpes / Other (specify______)

4. What do you know about the following contraceptive methods? (Only one answer is possible for each contraceptive method): I know it exists, but I don't know how to use it / I know how to use it, but I have never used it / I have used it and/or use it / I don't know it

Mehods: male condom / female condom / pill / IUD / diaphragm / patch / subcutaneous device / coitus interruptus / natural methods/calculation of fertile days / morning-after pill / abstinence / other method (specify_)

5. Which contraceptive methods protect against sexually transmitted infections? (multiple answers possible)

Methods: male condom / female condom / pill / IUD / diaphragm / patch / subcutaneous device / coitus interruptus / natural methods/fertile day calculation / morning-after pill / abstinence / other method (specify .........................) / none / don't know

*Behavior section*

6. Have you ever had sexual intercourse? Yes, sexual intercourse with penetration / Yes, sexual intercourse without penetration / No, no sexual intercourse

If you have had sexual intercourse, answer the next questions (7-9c):

7. At what age did you have your first full sexual intercourse? Age in years _____

8. At that time, what method did you and/or your partner use to avoid unwanted pregnancy and/or risk of infection/disease? Nothing / male condom / female condom / pill / IUD / diaphragm / patch / subcutaneous device / coitus interruptus / natural methods/fertile day calculation / morning-after pill / other method (specify ..............................................................) / don't know/don't remember

9. In the past 3 months, have you had sexual intercourse? (Do not consider your first sexual intercourse): Yes / No

9a. If yes, with how many people have you had sexual intercourse in the past 3 months? N. _____

9b. If yes, think about the last partner you had sex with: what method did you use to avoid an unwanted pregnancy or risk of infection/disease? Nothing / male condom / female condom / pill / IUD / diaphragm / patch / subcutaneous device / coitus interruptus / natural methods/fertile day calculation / morning-after pill / other method (specify ...............................) / don't know/don't remember

9c. If you answered nothing to the previous question, why? (multiple answers possible)

Options: It was an unplanned/unplanned relationship / The condom decreased the pleasure of intercourse / I didn't ask myself the question, I don't care / Condoms cost too much money / I am embarrassed to address the problem with the partner(s) / I trust the partner(s) / I know the partner(s), he/she is in good health / I think I am not at risk / The partner has refused / Other (specify ________)

10. What could be the reasons why young people use little or dislike using condoms? (multiple answers possible)

Options: Because they can be harmful at this age / Because of fear of being caught by parents / Because they take away from the spontaneity of relationships / Because they negatively affect performance / Because it is difficult to use them / Because it is embarrassing/difficult to obtain them / Because parents do not share their use of them / Because they cost too much / Because they are not known to exist / Because the partner prefers not to use it / Other (specify....................)

*Attitudes section*

11. Have you ever talked to anyone about any of the following topics? Never / Yes, superficially / Yes, in depth

Topics: Sentimental experiences / Sexuality and sexual relationships / Sexual consent* (*Clear and shared communication with a partner of the desire to participate or not to participate in a sexually involving situation, as well as to engage in certain sexual practices) / Sexually transmitted infections / Contraceptive methods

If for at least one of the items in the previous question the answer was Yes:

12. Who did you talk to about this? (multiple answers possible)

Options: With my mother / With my father / With my brothers/sisters / With friends/partners / With teachers / Other (specify ...............................) / Don't remember

*Information sources section*

13. Do you feel that your knowledge about sexuality and relationships is: None at all / Insufficient / Sufficient / Good

14. Have you ever actively sought information about sexuality and relationships? Yes, sometimes / Yes, often / No

14a. If yes, where (multiple answers possible)?

Options: In the family / From friends/partners / On the internet / On social media (instagram, tiktok, snapchat, etc.) / In magazines, books and/or TV / From a doctor / At school / Other (specify .........)

14b. If you answered "on the internet" to the previous question: what kind of source do you consult on the internet? Institutional/scientific sites on the topic / Generic sites / Forums and/or blogs / Pornographic sites / Other (specify ..............)

15. Do you think schools should ensure information on sexuality and relationships? Yes, starting with primary school / Yes, starting with lower secondary school / Yes, starting with upper secondary school / No / Don't know

15a. If yes, from whom would you like to receive information at school? From my teachers / From other teachers/experts inside the school / From expert personnel outside the school / Other (specify……….)

16. Have you ever participated in courses/meetings where the topic of sexuality and relationships was covered? Yes / No

16a. If yes, where (multiple answers possible)?

Options: At school / At health services (e.g., counseling centers; sexually transmitted infection centers - STI centers; dedicated outpatient clinics; health homes; etc.) / In the parish / At associations or at public events / Online / Other (specify.................................)

17. Which of these figures/entities have you approached? (multiple answers possible)

Options: Gynecologist / Andrologist / Psychologist / Center for sexually transmitted infections - STI center / Counseling Center / Other (specify.....................................................................) / None

*HPV Section*

18. Have you ever heard of HPV (papilloma virus)? (multiple answers possible)

Options: No / Yes, it causes genital condylomas / Yes, it causes several cancers in women only / Yes, it causes AIDS/HIV / Yes, causes several cancers in both women and men / Yes, causes hepatitis C / Yes, causes several cancers only in men

19. How is HPV (papilloma virus) transmitted? Through: (multiple answers possible)

Options: Handshake / Deep kiss / Contact between genitals / Vaginal intercourse / Anal intercourse / Oral intercourse / Use of shared sanitary facilities/public bathrooms / Use of contaminated objects (e.g., towels) / Blood transfusions / Exchange of syringes / Other (specify...................) / Don't know

20. Do you know that there is a vaccine for HPV (Papilloma Virus)? Yes / No

21. What diseases does the HPV (Papilloma Virus) vaccine prevent? (multiple answers possible)

Options: HIV/AIDS / Penile cancer / Genital condylomas / Cervical cancer / Syphilis / Tumor of the anus / Herpes / Other (specify........................................................) / Don't know

22. Have you been vaccinated for HPV (Papilloma Virus)? Yes / No / Don't know/don't remember

23. The choice to vaccinate/not to vaccinate: Was discussed in the family / Was left up to you / It was made by your parents / Don't know/don't remember / Other (specify..........................)

24. You did not get vaccinated why? (multiple answers possible)

Options: For fear of side effects / Because of lack of confidence in the vaccine / Because periodic Pap smears are sufficient / Because of fear of needles / Because of lack of information about it / Because not sexually active at vaccine call / Don't know / Other (specify.........................) / Not my decision

*Socio-demographic information section*

25. Gender: Female / Male / Other (if you want to specify..........................................................)

26. Age in completed years: ____ years

27. Country of birth ______________________________________

28. Municipality of residence __________________________________________________

29. Age of mother ____ years / don't know/not applicable

30. Father's age ____ years / don't know/not applicable

31. Citizenship of mother __________________ / don't know/not applicable

32. Citizenship of the father __________________ / don't know/not applicable

33. Highest educational qualification obtained by the mother: None / Primary school diploma / Lower secondary school diploma / Upper secondary school diploma / Bachelor's degree / Master's degree/research doctorate / Don't know/not applicable

34. Highest educational qualification obtained by father: None / Primary school diploma / Lower secondary school diploma / Upper secondary school diploma / Bachelor's degree / Master's degree/research doctorate / Don't know/not applicable

Thank you for participating.

**ESPRIT study - Questionnaire for parents**

Participant ID code: ______________

*Knowledge section*

1. Have you ever heard of sexually transmitted infections? Yes / No

2. How can sexually transmitted infections be transmitted? Through: (multiple answers possible)

Options: Handshake / Deep kissing / Contact between genitals / Vaginal intercourse / Anal intercourse / Oral intercourse / Use of sanitary facilities in communal/public bathrooms / Use of contaminated objects (e.g., towels) / Blood transfusions / Exchange of syringes / Other (specify..........................) / Don't know

3. In your opinion, can the following infections/diseases be transmitted through sexual intercourse? Yes / No / Don't know

Diseases: Viral hepatitis type B / Syphilis / Rubella / Chlamydia / Influenza / AIDS / Mediterranean anemia / Gonorrhea / Viral hepatitis type A / Papillomavirus infections / Diabetes / Genital herpes / Other (specify______)

4. What do you know about the following contraceptive methods? (Only one answer is possible for each contraceptive method): I know it exists, but I don't know how to use it / I know how to use it, but I have never used it / I have used it and/or use it / I don't know it

Options: male condom / female condom / pill / IUD / diaphragm / patch / subcutaneous device / coitus interruptus / natural methods/calculation of fertile days / morning-after pill / abstinence / other method (specify_)

5. Which contraceptive methods protect against sexually transmitted infections? (multiple answers possible)

Methods: male condom / female condom / pill / IUD / diaphragm / patch / subcutaneous device / coitus interruptus / natural methods/fertile day calculation / morning-after pill / abstinence / other method (specify .........................) / none / don't know

*Attitudes section*

6. Why, in your opinion, do young people not use condoms? (multiple answers possible)

Options: Because they think they can be harmful at this age / For fear of being caught by their parents / Because they take away from the spontaneity of relationships / Because it is difficult to use them / Because it is embarrassing/difficult to obtain them / Because parents do not believe in their use / Because they cost too much / Because their existence is unknown / Other (specify......)

7. Have you ever talked with your son/daughter about the following topics? Never/ Yes, superficially/ Yes, in depth

Topics: Sentimental experiences / Sexuality and sexual relationships / Sexual consent* (*clear and shared communication with a partner of the desire to participate or not to participate in a sexually involving situation, as well as to engage in certain sexual practices) / Sexually transmitted infections / Contraceptive methods

If for at least one of the items in the previous question the answer was yes:

8. With the support of what tools? Books / Movies/documentaries / Brochures / Other (specify............) / None

If for at least one of the items in question 7 the answer was negative:

9. For what reason? Doesn't feel prepared / It is embarrassing / It is not my job / My son/daughter has never asked for this / My son/daughter does not want to deal with these topics

10. Who else have you talked to about this? (multiple answers possible)

Options: With work colleagues / With your son/daughter's teachers / With the paediatrician / With the general practitioner / With other medical specialists / Other (specify ................) / Do not remember

*Information sources section*

11. Do you feel that your knowledge about sexuality and sexual relationships is: None at all / Insufficient / Sufficient / Good

12. Have you ever actively sought information about sexuality and relationships? Yes, sometimes / Yes, often / No

12a. If yes, where (multiple answers possible)?

Options: In the family / From friends / On the internet / On social media (instagram, tiktok, snapchat, etc.) / In magazines, books and/or TV / From a doctor / At work / Other (specify ............)

12b. If you answered "on the internet" to the previous question what type of source did you consult on the internet? Institutional/scientific sites on the topic / Generic sites / Forums and/or blogs / Other (specify ..............)

13. Do you think schools should ensure information on sexuality and relationships? Yes, starting with primary school / Yes, starting with lower secondary school / Yes, starting with upper secondary school / No / Don't know

13a. If yes, from whom would you like your son/daughter to receive information at school? From his or her teachers / From other teachers/experts inside the school / From expert personnel outside the school / Other (specify...................)

14. Have you ever participated in courses/meetings aimed at parents where the topic of sexuality and sexual relationships was discussed? Yes / No

14a. If yes, where? In the workplace / At my child's school / At health services (e.g., counseling centers; sexually transmitted infection centers - STI centers; dedicated outpatient clinics; health homes; etc.) / At a parish / At associations or at public events / Online / Other (specify..................)

15. To your knowledge, to which of these figures/entities has your son/daughter turned to? (multiple answers possible)

Options: Gynecologist / Andrologist / Psychologist / Center for sexually transmitted infections - STI center / Counseling Center / Other (specify.............................) / None / Don't know

*HPV section*

16. Have you ever heard of HPV (papilloma virus)? (multiple answers possible)

Options: No / Yes, it causes genital condylomas / Yes, it causes several cancers in women only / Yes, it causes AIDS/HIV / Yes, causes several cancers in both women and men / Yes, causes hepatitis C / Yes, causes several cancers only in men

17. How is HPV (papilloma virus) transmitted? Through: (multiple answers possible)

Options: Handshake / Deep kissing / Contact between genitals / Vaginal intercourse / Anal intercourse / Oral intercourse / Use of shared sanitary facilities/public bathrooms / Use of contaminated objects (e.g., towels) / Blood transfusions / Exchange of syringes / Other (specify.............................................) / Don't know

18. Do you know that there is a vaccine against HPV (Papilloma Virus)? Yes / No

19. What diseases does the HPV (Papilloma Virus) vaccine prevent? (multiple answers possible)

Options: HIV/AIDS / Penile cancer / Genital condylomas / Cervical cancer / Syphilis / Tumor of the anus / Herpes / Other (specify........................................................) / Don't know

20. Have you vaccinated or do you plan to vaccinate your/your son/daughter for HPV (Papilloma Virus)? Yes / No

If the answer to the previous question is No:

21. Why have you not had your son/daughter vaccinated or do not plan to have your son/daughter vaccinated? (multiple answers possible)

Options: Out of fear of side effects / Because of lack of confidence in the vaccine / Because periodic Pap smears are sufficient / Out of fear of needles / Because of lack of information about it / Because not sexually active at vaccine call / Don't know / Other (specify.................................)

22. The choice to vaccinate/not vaccinate your son/daughter: Was discussed in the family / Was left up to you / Was taken by you and your husband/partner or wife/partner / Does not know/does not remember

*Socio-demographic information section*

23. Gender: Female / Male / Other (if you want to specify.................................................)

24. Age in completed years: ___years

25. Country of birth ______________________________________

26. Municipality of residence __________________________________________________

27. Citizenship ______________________________________

28. Highest educational qualification obtained: None / Primary school diploma / Secondary lower school diploma / Secondary upper school diploma / Bachelor's degree / Master's degree/research doctorate / Other (specify____________________)

Thank you for participating.

**ESPRIT study - Questionnaire for teachers**

Participant ID code: ______________

*Knowledge section*

1. Have you ever heard of sexually transmitted infections? Yes / No

2. How can sexually transmitted infections be transmitted? Through: (multiple answers possible)

Options: Handshake / Deep kissing / Contact between genitals / Vaginal intercourse / Anal intercourse / Oral intercourse / Use of sanitary facilities in communal/public bathrooms / Use of contaminated objects (e.g., towels) / Blood transfusions / Exchange of syringes / Other (specify..........................) / Don't know

3. In your opinion, can the following infections/diseases be transmitted through sexual intercourse? Yes / No / Don't know

Diseases: Viral hepatitis type B / Syphilis / Rubella / Chlamydia / Influenza / AIDS / Mediterranean anemia / Gonorrhea / Viral hepatitis type A / Papillomavirus infections / Diabetes / Genital herpes / Other (specify______)

4. What do you know about the following contraceptive methods? (Only one answer is possible for each contraceptive method): I know it exists, but I don't know how to use it / I know how to use it, but I have never used it / I have used it and/or use it / I don't know it

Options: male condom / female condom / pill / IUD / diaphragm / patch / subcutaneous device / coitus interruptus / natural methods/calculation of fertile days / morning-after pill / abstinence / other method (specify_)

5. Which contraceptive methods protect against sexually transmitted infections? (multiple answers possible)

Methods: male condom / female condom / pill / IUD / diaphragm / patch / subcutaneous device / coitus interruptus / natural methods/fertile day calculation / morning-after pill / abstinence / other method (specify .........................) / none / don't know

*Attitudes section*

6. Why, in your opinion, do young people not use condoms? (multiple answers possible)

Options: Because they think they can be harmful at this age / For fear of being caught by their parents / Because they take away from the spontaneity of relationships / Because it is difficult to use them / Because it is embarrassing/difficult to obtain them / Because parents do not believe in their use / Because they cost too much / Because their existence is unknown / Other (specify......)

7. Have you ever talked with your students about the following topics? Never/ Yes, superficially/ Yes, in depth

Topics: Sentimental experiences / Sexuality and sexual relationships / Sexual consent* (*clear and shared communication with a partner of the desire to participate or not to participate in a sexually involving situation, as well as to engage in certain sexual practices) / Sexually transmitted infections / Contraceptive methods

If for at least one of the items in the previous question the answer was yes:

8a. Under what circumstance did you talk to your/your students? During one of my classes / During a dedicated class meeting / During extracurricular activities / During a personal/private conversation with a student / Other (specify......................)

8b. With the support of what tools? Books / Films/documentaries / Brochures / Other (specify.........) / None

If for at least one of the items in question 7 the answer was negative:

9. For what reason? Don't feel prepared / It is embarrassing / It is not my job / Student(s) never asked / Student(s) do not want to deal with these topics

10. Who else have you talked to about this? (multiple answers possible)

Options: With colleagues / With the parents of student(s) / With a paediatrician / With a general practitioner / With other medical specialists / Other (specify .......................) / Do not remember

*Information sources section*

11. Do you feel that your knowledge about sexuality and sexual relationships is: None at all / Insufficient / Sufficient / Good

12. Have you ever actively sought information about sexuality and relationships? Yes, sometimes / Yes, often / No

12a. If yes, where (multiple answers possible)? In the family / From friends / On the internet / On social media (instagram, tiktok, snapchat, etc.) / In magazines, books and/or TV / From a doctor / At work / Other (specify ...................)

12b. If you answered "on the internet" to the previous question what type of source did you consult on the internet? Institutional/scientific sites on the topic / Generic sites / Forums and/or blogs / Other (specify ............)

13. Do you think schools should ensure information on sexuality and relationships? Yes, starting with primary school / Yes, starting with lower secondary school / Yes, starting with upper secondary school / No / Don't know

13a. If yes, from whom do you think pupils should receive this information in school? (multiple answers possible)

Options: From teachers / From other teachers/experts inside the school / From expert personnel outside the school / Other (specify.........................)

14. Have you ever participated in courses/meetings aimed at teachers where the topic of sexuality and sexual relationships was covered? Yes / No

14a. If yes, where? In the workplace / At health services (e.g., counseling centers; sexually transmitted infection centers - STI centers; dedicated outpatient clinics; health homes; etc.) / In the parish / At associations or at public events / Online / Other (specify....................)

*HPV section*

15. Have you ever heard of HPV (papilloma virus)? (multiple answers are possible)

Options: No / Yes, it causes genital condylomas / Yes, it causes several cancers in women only / Yes, it causes AIDS/HIV / Yes, causes several cancers in both women and men / Yes, causes hepatitis C / Yes, causes several cancers only in men

16. How is HPV (papilloma virus) transmitted? Through: (multiple answers are possible)

Options: Handshake / Deep kissing / Contact between genitals / Vaginal intercourse / Anal intercourse / Oral intercourse / Use of shared sanitary facilities/public bathrooms / Use of contaminated objects (e.g., towels) / Blood transfusions / Exchange of syringes / Other (specify.............................................) / Don't know

17. Do you know that there is a vaccine against HPV (Papilloma Virus)? Yes / No

18. What diseases does the HPV (Papilloma Virus) vaccine prevent? (multiple answers possible)

Options: HIV/AIDS / Penile cancer / Genital condylomas / Cervical cancer / Syphilis / Tumor of the anus / Herpes / Other (specify........................................................) / Don't know

19. Have you ever talked about HPV (Papilloma Virus) vaccination to your/your pupils? (multiple answers possible) Yes / No

*Socio-demographic information section*

20. Gender: Female / Male / Other (if you want to specify...............................................)

21. Age in completed years: ____ years

22. Country of birth _______________________________________________________

23. Municipality of residence __________________________________________________

24. Citizenship __________________________________

25. Highest educational qualification obtained: Upper secondary school diploma / Bachelor's degree / Master's degree/research doctorate / Other (specify________________________________)

26. What subject do you teach? Please specify.......................................................................

27. Years of work experience in the school: __ years

Thank you for participating
